# The division of labour between community medicine distributors influences the reach of mass drug administration: A cross-sectional study in rural Uganda

**DOI:** 10.1371/journal.pntd.0007685

**Published:** 2019-09-04

**Authors:** Goylette F. Chami, Narcis B. Kabatereine, Edridah M. Tukahebwa

**Affiliations:** 1 Department of Pathology, University of Cambridge, Cambridge, United Kingdom; 2 Big Data Institute, Clinical Trial Service Unit and Epidemiological Studies Unit, Nuffield Department of Population Health, University of Oxford, Oxford, United Kingdom; 3 Vector Control Division, Bilharzia and Worm Control Programme, Uganda Ministry of Health, Kampala, Uganda; University of California Berkeley, UNITED STATES

## Abstract

**Background:**

Despite decades of community-based mass drug administration (MDA) for neglected tropical diseases, it remains an open question as to what constitutes the best combination of community medicine distributors (CMDs) for achieving high (>65%/75%) treatment rates within a village.

**Methods:**

Routine community-based MDA was evaluated in Mayuge District, Uganda. For one month, we tracked 6,148 individuals aged 1+ years in 1,118 households from 28 villages. Praziquantel, albendazole, and ivermectin were distributed to treat *Schistosoma mansoni*, lymphatic filariasis, and soil-transmitted helminths. The similarity/diversity between CMDs was observed and used to predict the division of labour and overall village treatment rates. The division of labour was calculated by dividing the lowest treatment rate by the highest treatment rate achieved by two CMDs within a village. CMD similarity was measured for 16 characteristics including friendship network overlap, demographic and socioeconomic factors, methods of CMD selection, and years as CMD. Relevant variables for MDA outcomes were selected through least absolute shrinkage and selection operators with leave-one-out cross validation. Final models were run with ordinary least squares regression and robust standard errors.

**Results:**

The percentage of individuals treated with at least one drug varied across villages from 2.79–89.74%. The only significant predictor (p-value<0.05) of village treatment rates was the division of labour. The estimated difference between a perfectly equal (a 50–50 split of individuals treated) and unequal (one CMD treating no one) division of labour was 39.69%. A direct tie (close friendship) between CMDs was associated with a nearly twofold more equitable distribution of labour when compared to CMDs without a direct tie.

**Conclusions:**

An equitable distribution of labour between CMDs may be essential for achieving treatment targets of 65%/75% within community-based MDA. To improve the effectiveness of CMDs, national programmes should explore interventions that seek to facilitate communication, friendship, and equal partnership between CMDs.

## Author summary

Community-based mass drug administration (MDA) uses volunteers within at-risk communities to distribute preventive chemotherapies *en masse* for neglected tropical diseases. Treatment rates achieved by community medicine distributors (CMDs) vary widely and can undermine morbidity control. We studied routine community-based MDA in 28 villages near Lake Victoria in Uganda. There were two CMDs per village who were tasked with moving from home-to-home to administer praziquantel, albendazole, and ivermectin for schistosomiasis, lymphatic filariasis, and soil-transmitted helminths. We observed treatment outcomes for 6,148 eligible individuals aged 1+ years. Here we identified the best combination of CMD characteristics for achieving high village-level treatment rates. We found that a more equal division of labour (e.g. 50–50 split between how many people each CMD treated) was associated with higher treatment rates when compared to CMDs with an unequal division of labour (e.g. one CMD treating no one). CMDs who were friends were more likely to have a division of labour that was nearly twofold more equal than CMDs who were not friends. The similarity of CMDs with respect to network, demographic, or socioeconomic characteristics did not influence village treatment rates. National programmes should explore interventions that seek to facilitate communication, friendship, and equal partnership between CMDs.

## Introduction

For public interventions, the first-informed individuals influence the spread of information and uptake within the target population [1]. Understanding who should be the first-informed individuals or the deliverers of an intervention is a widespread challenge for any area of public policy, but in particular for global health programmes [2–4]. Little is known about how best to introduce and to maintain global health programmes in resource-poor settings where access to formal medical care and health-seeking behaviours are limited. Effective global health programmes rely on identifying the appropriate starting points for an intervention, e.g. who should deliver drugs, who should be treated first, and who should act as health promoters.

One successful and extensively used model for identifying the starting points for global health programmes is mass drug administration (MDA) [5]. MDA is the blanket, diagnosis-free distribution of single dose preventive chemotherapies to individuals within at-risk areas for neglected tropical diseases (NTDs) [6]. The frequency and implementation of MDA is determined by the prevalence of infection within a geographical catchment, school, or community and varies by disease [6]. Several methods of MDA implementation exist, utilizing communities, primary schools, or child health days. The most common method of implementation is through community-based MDA, which is used to treat schistosomiasis, lymphatic filariasis, trachoma, and onchocerciasis with some communities also benefiting from the treatment of soil-transmitted helminths (STHs) because of coendemicity with lymphatic filariasis. To promote local ownership of MDA, national programmes instruct individuals within NTD-endemic areas to select local community medicine distributors (CMDs) through open, community-wide meetings [7]. CMDs serve as volunteers, apart from the reimbursement for travel costs to attend annual training sessions, and are tasked with either moving from home-to-home (e.g. schistosomiasis) [8] or with mobilizing individuals to retrieve drugs from a central post (e.g. lymphatic filariasis) [9]. Progress towards NTD control, including community-based MDA, has been proposed as a platform for measuring access to universal health coverage [10]. In 2017, nearly 1/3^rd^ of school-aged children, who are included in the World Health Organization (WHO) Roadmap for NTDs [11] and require preventive chemotherapies for schistosomiasis or STHs remained untreated [12]. For example, after 10 years of community-based MDA in Mayuge District, Uganda, CMDs treated only 56.66% of eligible individuals with at least one drug for schistosomiasis, lymphatic filariasis, or STHs [13]. Therefore, a better understanding is needed of how to increase the effectiveness of CMDs.

The context in which CMDs have been studied in order to improve MDA includes 1) how best to alleviate the opportunity costs of time volunteered [14, 15], 2) how to reduce capacity constraints resulting from a limited number of CMDs [14, 16, 17], 3) the impact of financial or in-kind incentives for CMDs [18], 4) the role of knowledge, attitudes, and practice as well as available health system support in promoting CMD motivation [19], 5) the social biases that manifest in the CMD’s decision on whom to treat [13, 20], and 6) the personal characteristics of CMDs that determine their performance during MDA [21]. Despite the wide variation of treatment rates across communities [4], there is a limited understanding from both national MDA programmes and communities of how and whether CMDs should be replaced before they choose to resign, and specifically of what combination of CMDs is best for achieving the highest treatment rates. Two aspects of CMD selection have been studied and associated with increased treatment rates: exploiting local social network structures to choose well-placed CMDs [4, 22] and including CMDs with diverse kinship affiliations so that a CMD treats only individuals with a shared clan membership [16, 17]. The kinship studies [16, 17] identify similarity between CMDs and MDA recipients rather than measure the similarity between CMDs, and do not consider network or socioeconomic similarity. Thus, it remains an open question as to if/how the similarity of CMDs affects MDA outcomes.

To identify the best combination of CMDs, there is a need to understand how network, demographic, and socioeconomic similarity between CMDs affects their performance during MDA. Similarity has been widely shown elsewhere to determine peer effects, i.e. how one person influences another person (either directly or indirectly) [23–28]. Yet, how CMDs influence one another or how shared CMD affiliations affect MDA, to our knowledge, has not been studied. Here we conduct the first analysis of CMD similarity by comparing the networks and personal attributes of CMDs to identify what combination of CMDs best facilitates the reach of MDA. Moreover, to further delve into peer effects, we provide the first study of how CMD similarity influences the division of labour between CMDs. We answer the following question. How does CMD similarity affect the division of labour and treatment rates achieved during MDA?

## Methods

### Ethics statement

This study was reviewed and approved by the Uganda National Council of Science and Technology (SS4077), and the University of Cambridge School of Humanities and Social Sciences (HSSREC2016.6). Written informed consent was obtained from all respondents. For respondents who indicated they were unable to write or who preferred to provide fingerprints, verbal informed consent and a fingerprint signature were obtained.

### MDA & CMD selection

Using methods described and validated in Chami *et al*. [4, 20], routine community-based MDA was tracked in 31 villages in Mayuge District, Uganda from mid-July to mid-August 2016. The study area predominantly comprises fishing villages along Lake Victoria, which are hyperendemic (>50% prevalence) with *Schistosoma mansoni* [29]. To remove administrative barriers that may delay the start of MDA, researchers provided local District Vector Control Officers—the individuals responsible for routinely training CMDs—with cars to start MDA within three days in July for all study villages. Study surveys were conducted after one month of MDA. Preventive chemotherapies were only available from the community-based MDA programmes during the study period. Two CMDs were tasked with approaching all households, i.e. moving door-to-door, and administering preventive chemotherapies. There were no limits on treatment rates achievable by CMDs due to insufficient medicine supplies. Researchers provided the Vector Control Officers with enough pills/tablets for all CMDs to treat all eligible individuals within their villages. Survey teams conducted surprise checks of CMD homes after the one-month MDA tracking and pills/tablets for all medicines remained with all CMDs. Praziquantel was distributed to treat school-aged children and adults (individuals aged 5+ years) for potential infections with *S*. *mansoni*. Albendazole and ivermectin were administered to treat school-aged children and adults (all individuals aged 5+ years old) for potential lymphatic filariasis infections. Due to hookworm endemicity, albendazole was provided to pre-school aged children, school-aged children and adults (all individuals aged 1+ years old), although albendazole was not donated for treating hookworm through community-based MDA [29]. The most common method of MDA implementation for schistosomiasis and STHs in Uganda is the distribution of medicines through primary schools, i.e. excluding adults for treatment and using schoolteachers as distributors instead of CMDs [8]. The high prevalence of *S*. *mansoni* infections and the endemicity of lymphatic filariasis enabled community-wide treatment for schistosomiasis and STHs, respectively. When lymphatic filariasis treatment stops in our study area then community-wide treatment may stop for STHs.

MDA was community-based as opposed to community-directed in that communities did not lead the design of MDA, which was completed by the national programmes. Communities selected CMDs, but did not choose the dates, time period, or method of distribution for MDA. Communities also were not formally involved in the monitoring of CMDs, which was the task of the District Vector Control Officers. The national MDA programme instructed communities to select systematically two CMDs through a community-wide meeting and to choose individuals who were literate and able to fill in NTD registers. Communities also were encouraged to have gender balance between CMDs, i.e. one female and one male CMD per village. No other instructions for the selection or replacement of CMDs were provided by the national MDA programme. Communities did not strictly follow national recommendations. Village leaders (local government members or village health team members) directly selected more than half of the CMDs instead of holding community-wide meetings [21].

### Participant sampling

Systematic random sampling of households was conducted [21]. Village registers of households—ordered by year of settlement—were used to select 40 households per study village. Household heads and lead wives were interviewed to provide information on all members of the household aged 1+ years—the minimum criteria for MDA eligibility. In addition to the systematic random sampling, all CMDs and their household heads were interviewed. Households of CMDs only were included in the calculation of treatment outcomes if selected by chance through the systematic random sampling.

### Treatment outcomes

Using a structured questionnaire [21], two sets of treatment outcomes were examined for participants who were selected through systematic random sampling: village treatment rates and the division of labour between CMDs. Village treatment rates comprised the overall level of treatment within a village, i.e. the work of both CMDs, and were calculated at both the individual and household levels. Treatment responses were recorded by an independent team of surveyors who conducted surprise visits to villages after one month of undisturbed MDA, as described in Chami *et al*. [4, 20]. At the individual level, treatment rates were measured as the percentage of eligible individuals who were offered and had ingested at least one drug of praziquantel, albendazole, or ivermectin. This indicator most closely aligns with the WHO’s indicator of surveyed coverage [4]. We used a conservative measure of treatment with at least one drug to reduce the dimensionality of the analysis (number of models run) and to account for endogeneity that arises with individual drug outcomes, i.e. drug-specific treatment rates are strongly positively correlated [21]. At the household level, treatment rates were measured as the percentage of households with at least one eligible person who was offered and had ingested at least one drug of praziquantel, albendazole, or ivermectin. Household-level treatment rates were of interest as the Uganda MDA programme instructed CMDs to move from home-to-home within our study area and treatment rates for individuals are strongly positively correlated within a home [20]. Also, household-level treatment rates represent the percentage of homes approached by CMDs [4]. WHO disease-specific treatment targets include treatment of 75% of eligible individuals with praziquantel for schistosomiasis and albendazole for hookworm, and 65% of eligible individuals with albendazole plus ivermectin for lymphatic filariasis. As a note, treatment outcomes were determined by drug delivery efforts from CMDs since only less than 1% of MDA recipients refused to ingest offered medicines [21]. Hence, in our study, the offer and ingestion of medicine (treatment) also can be thought of as indicative of evidence of contact with CMDs.

### Division of labour

We undertook a proof-of-principle investigation into the relevance of the division of labour for predicting village treatment rates, which are used to assess progress towards WHO treatment targets [30]. To develop the first measure of the division of labour for CMDs, we sought a simple indicator that 1) did not interfere with routine MDA, 2) captured the primary objective of MDA, i.e. maximizing the number of people treated, and 3) could be applied in various geographical or social contexts. In this respect, the division of labour was outcome-based and focused on the number of people treated by each CMD. Importantly, neither the national MDA programme nor local health facilities provided instructions to CMDs for dividing labour. The national MDA programme only indicated to CMDs that they should treat all eligible individuals within their village. Hence, CMDs were not pre-allocated households or geographical areas of a village. CMDs did not hold discussions with their communities about the division of labour. Consequently, we assumed here that how best to divide labour to meet programmatic goals was the sole decision of CMDs.

The entire village was considered for assessing the labour of each CMD. In Uganda, the village is the lowest administrative unit; there are no further formal subdivisions that could have been exploited for the division of labour. Moreover, no intra-community spatial divisions were considered due to the small size of study villages and previously shown irrelevance of the number of homes for explaining village treatment rates in our study area [4]. On average, there were only 238 homes per village (range 87–535 homes). Concerning spatial aspects, the village ecology, such as the number of roads or swamps, has been shown to be uninformative for MDA in our study area [4]. The spatial spread/diameter of the study villages also has been shown to be unrelated to village treatment rates [4]. The furthest distance in metres between two homes has been shown to be on average only 1.26 km (std. dev. 428.29 m) [4]. The mean distance between two homes within our study villages has been measured at 400.11 m (std. dev. 142.27 m) [4].

For the division of labour, the percentage of eligible individuals or eligible households treated by each CMD was examined. For drugs offered to eligible individuals, the household respondent provided the name of the CMD who offered treatment. All respondents knew who treated whom. Few eligible individuals (<12%) were offered treatment by both CMDs, which included either CMDs separately approaching the same individual or both CMDs being present at the same time to treat the same individual. More detailed methods on the calculation/attribution of individual CMD treatment rates are provided in Chami *et al*. [21].‘Treated’ was defined as described for the village-level treatment rates. The division of labour was calculated as a ratio of treatment rates for the two CMDs in each village. For the two CMDs, the lowest treatment rate was divided by the highest treatment rate to create a normalized village-level outcome. The division of labour was an indicator from 0–1 where 0 was a perfectly unequal division of labour (one CMD treated no one) and 1 was a perfectly equal division of labour (CMD treatment rates were equal). There were no villages where both CMDs treated no one. The division of labour was calculated for treatment rates at both the individual and household levels. Although MDA consists of separate tasks such as registering households, sensitizing individuals, and mobilizing the community, CMDs in our study area perform these tasks whilst they treat individuals [4, 13, 21]. Thus, in our study context, the division of labour for treatment outcomes also represents the division of labour dedicated to diverse MDA tasks.

### Networks

CMDs were interviewed and asked to provide the names of their close friends, using the following structured prompt [4, 13, 22].

“Please tell me the clan name first then the second name of up to 10 people that are very close friends to you. You should feel comfortable to turn to this person to borrow tools for fishing or farming without paying. A close friend is also someone that you see frequently. You should trust this person for advice. Do not name anyone in your household. Provide the names in the order of who is your closest friend first. Only name people in your village.”

The individuals named as close friends by CMDs also were interviewed. The friends of CMDs were provided with a list of names, which included all individuals who were named by both CMDs as well as the names of the CMDs. Friends of CMDs were then asked to indicate with whom they had close friendships. Hence, CMDs could belong to the same network component, i.e. a path could exist between the two CMDs, due to either a friendship between CMDs or a friendship between the friends of CMDs. Moreover, a CMD could have more than 10 ties due to the friends of the other CMD naming the CMD of interest. All friendship networks were analyzed as undirected; if an individual was named or had named someone then there was a tie between those two individuals.

### Variables

Sixteen indicators of similarity between the two CMDs in each village were examined, which included three network characteristics and 13 personal attributes. For network similarity (structural equivalence), three variables that captured both direct and indirect ties were calculated using NetworkX in Python v2.7 [31]. A direct tie was a binary indicator of friendship between CMDs. The Jaccard index captured indirect ties between CMDs and the similarity of their network neighbourhood, i.e. common friends. It was calculated as the number of common friends divided by the total number of friends across both CMDs. To gain insight into the cohesion between CMDs and to account for the fact that influence between CMDs may travel further than two network steps (beyond common neighbours) [27, 32], the minimum node cut was calculated. The minimum number of nodes (friends) that would need to be removed from the network to disconnect CMDs, i.e. to remove all paths between CMDs, was counted then normalized by dividing by the total number of friends for both CMDs. A comparison of direct ties versus indirect ties was of interest to understand by what means could peer effects occur between CMDs [24]. With direct ties, peer effects occur due to an existing channel of communication between two individuals [28]. Alternatively, indirect ties have been shown to influence two individuals of interest through either competition or comparison [25]. For competitive influences, it has been shown that two individuals vie for the attention of the same friends (due to the overlapping friendship group) and this competition is what drives similar behaviours [25, 26]. Alternatively, indirect ties may signal shared friendships that are used as a reference for behaviours, i.e. an individual compares themself to their group of friends and two individuals with shared friends will compare themselves to the same group [4, 26, 27].

CMDs were interviewed using a structured questionnaire [21] in order to observe 13 personal attributes. The method of CMD selection was recorded as a categorical variable and included nominations from a community-wide meeting, the village health team, or a local council (village government) member. The total number of years as a CMD was noted. Eleven demographic and socioeconomic characteristics were observed. Age was rounded to the nearest year. Gender was a binary variable and equal to one if the CMD was female. Education was measured as a categorical variable to represent the highest level of education attained and included primary school, secondary school, or post-secondary school diplomas. Binary indicators for majority tribe and religion were equal to one if the CMD belonged to the majority tribe or religion of their village, respectively. Occupation was a categorical variable that included values for farmer, fisherman/fishmonger, and ‘other’ jobs; occupation was coded to capture the main occupations in the study area [29]. Formal status was a binary indicator that was equal to one if the CMD was a religious/tribe/clan leader, on the beach management committee, or a member of the local council (village government). Two binary indicators of preventative health behavior were measured using WHO and United Nations International Children’s Emergency Fund (UNICEF) guidelines [33]. A CMD belonged to a household that used a protected water source if drinking water was retrieved from piped water, village taps, boreholes, or protected wells. Private home latrine ownership included only covered pit latrines with privacy. Home quality score was the total sum of scores (min. 3, max. 12) for the floor, walls, and roof materials (four for each category, ranked 1–4) [13]. The ‘years in village’ was a count of the total years since the CMD’s household had settled in the current village.

To calculate attribute similarity between CMDs, binary indicators were constructed for all 13 CMD attributes, including MDA-related variables, and equal to one if CMDs were similar [34]. For all binary or categorical variables, if CMDs shared the same value/category then the attribute indicator was equal to one. For the number of years settled in the village, CMDs were coded as similar if their years of settlement were within +5/-5. For years as CMD, age, and home quality score variables, CMDs were classified as similar if their values were within +3/-3. In addition to the CMD similarity indicators, we accounted for variation in village and network size [4, 27]. The natural log of total homes in the village and the natural log of the average CMD degree (total friendship ties for each CMD) were calculated.

### Statistical analysis

Statistical analyses were completed at the village level and conducted in *R* v3.2.3 and Stata v13.1. With a limited number of village observations and no previous work on CMD similarity, we employed an unsupervised approach. Leave-one-out-cross-validation (LOOCV) with least absolute shrinkage and selection operators (LASSO) [35, 36] were used to select the predictors of the division of labour and village treatment rates. This approach is a commonly used method for dimension reduction in statistical analyses. LOOCV LASSO was run with simple ordinary least squares (OLS) [35, 36]. For the selection of predictors for the division of labour, all 16 CMD variables as well as the village and network sizes described in the previous section were candidates. In addition to these 18 variables, the division of labour was included for the selection of predictors of village treatment rates. The predictors that were selected through LOOCV LASSO were then entered in OLS regressions with robust standard errors [37]. To test for potential endogeneity of the division of labour and village treatment rates, i.e. an incorrectly specified direction of association where village treatment rates may determine the division of labour or more generally an association of the two outcome equations through the error terms, a Durbin-Wu-Hausman test was conducted and seemingly unrelated regressions were run [38]. For the Durbin-Wu-Hausman test, no evidence was found to indicate that the two outcome equations were correlated (F-stat = 2.73, p-value = 0.111 for individual-level outcomes and F-stat = 0.74, p-value = 0.399 for household-level outcomes). Similarly, no support for simultaneous equations was found from the seemingly unrelated regressions (Chi^2^ = 0.498, p-value = 0.481 for individual-level outcomes, and Chi^2^ = 0.602, p-value = 0.438 for household-level outcomes). Thus, separate OLS regressions were run. LOOCV was run for both the selection of the predictors and for the final models.

### Final sample studied

For the statistical analyses, three villages (IDs 20, 24, 30) were not included because one CMD in each of those villages was missing network information. Thus, 56 CMDs from 28 villages had complete data. For the target population, two households were excluded due to having no eligible individuals for MDA or missing information regarding treatment. In total, 1,118 households and 6,148 eligible individuals within those households were observed.

## Results

### Treatment & the division of labour

In 28 villages, 47.87% (2943/6148) and 24.77% (1523/6148) of eligible individuals were treated with at least one drug and all three drugs, respectively. Only 56.71% (634/1118) of households had at least one eligible person treated with praziquantel, albendazole, or ivermectin. Treatment rates achieved by individual CMDs ranged from 0–84.25% (std. dev. 22.09%) and 0–87.50% (std. dev. 23.49%) for individuals and households, respectively (Obs. 56). Village treatment rates also varied widely within the study area. The percentage of eligible individuals treated in each village varied from 2.79–89.74% (std. dev. 26.15%). Similarly, the percentage of households with at least one eligible person treated ranged from 7.50–97.50% (std. dev. 25.10%). WHO treatment targets for each disease were not necessarily met when village treatment rates (as measured here) met WHO-recommended levels. For schistosomiasis and hookworm, five communities had village treatment rates of at least 75% (Village IDs 1,14,18,21,31) and three communities had praziquantel and albendazole treatment rates of at least 75% (Village IDs 1,18,31). For lymphatic filariasis, 10 communities (Village IDs 1,2,12,14,17,18,21,25,26,31) had village treatment rates of at least 65% yet only three communities had both albendazole and ivermectin treatment rates of at least 65% (Village IDs 1, 18, 31). Therefore, only 10.71% (3/28) of villages met WHO-recommended treatment targets for each disease.

The division of labour across villages was highly unequal. The average division of labour for both the percentage of individuals and households treated was 0.327 (std. dev. 0.277 for individuals and 0.285 for households). In other words, when two CMDs were compared within the same village, one CMD treated on average only one third as many people or households as their counterpart. Wide variation in the division of labour was observed. The divisions of labour for individual and household level outcomes ranged from perfectly unequal to nearly a perfectly equal 50–50 split (range 0–0.967, std. dev. 0.278 for individuals; and 0–0.957, std. dev. 0.285, for households). There were six villages with one CMD who treated no one. Despite the wide variation in the division of labour, high inequality between CMDs was most common. For example, for the percentage of individuals treated, 75% of villages had a division of labour between CMDs where one CMD treated twice as many people as the other CMD. The unequal division of labour was not due to both CMDs treating few people, i.e. one CMD treating marginally more individuals (e.g. 10% versus 5%). The average absolute difference in the percentage of eligible individuals treated between CMDs within a village was 30.23% (std. dev. 20.85%).

### CMD similarity

A summary of personal attributes of CMDs, similarities between CMDs, and village sizes are presented in Tables 1–3. Within the study area, there was no single characteristic that was shared by all CMDs. When two CMDs within the same village were compared by personal/observable characteristics, CMDs were most often similar with respect to preventative health behaviours and socioeconomic status. A large majority (81.14%) of villages had two CMDs with the same ownership status of private home latrines; in all but one of these villages (22/23) both CMDs owned a private home latrine. In 75.00% of villages, both CMDs had the same formal status (where 4/21 villages had both CMDs with formal status) and similar home quality scores. Approximately 50% or more of villages had two CMDs who differed with respect to gender, educational attainment, membership in the majority tribe, and the number of years settled in the village. There were 25.00% (7/28) and 17.86% (5/28) of villages, respectively, where CMDs were either both females or both males. With respect to MDA-related characteristics, only 53.57% of villages had CMDs who were selected through the same means and only 42.86% of villages had two CMDs who had volunteered for MDA for a similar number of years. CMDs selected in the same manner were not necessarily selected through community-wide meetings. Only 40.00% (6/15) of villages who selected both CMDs in the same manner did so through community-wide meetings whilst the remainder of those villages had both CMDs selected by a member of the local council (village government).

**Table 1:** Personal attributes of community medicine distributors.

Variable		*Proportion* Mean	*Frequency* Std. Dev.
Selected through community-wide meeting	56	*41*.*07*	*23*
Selected by local council member (village government)	56	*53*.*57*	*30*
Selected by village health team member	56	*5*.*36*	*3*
Years as CMD	56	8.82	4.57
Age	56	42.38	10.09
Female	56	*53*.*57*	*30*
Education	56	8.32	1.95
Majority tribe	56	*51*.*79*	*29*
Majority religion	56	*50*.*00*	*28*
Fishermen/fishmonger	56	*8*.*93*	*5*
Farmer	56	*73*.*21*	*41*
Formal status	56	*26*.*79*	*15*
Use protected drinking water sources	56	*80*.*36*	*45*
Private home latrine ownership	56	*87*.*50*	*49*
Home quality score	56	9.59	2.77
Years settled in village	56	21.95	10.56

**Table 2:** Village size and friendship network properties.

Variables	Obs.	Mean	Std. Dev.	Min	Max
Total homes	28	238.429	123.419	87	535
LN(total homes)	28	5.357	0.484	4.466	6.282
Avg. No. people per household in each village^a^	28	5.61	0.69	4.08	6.93
Est. No. people per village^b^	28	1338.25	724.45	423.80	3557.75
In-degree: total friends named by CMD = outgoing ties	56	4.768	1.513	2	9
Degree: total friends = outgoing + incoming ties	56	8.429	2.295	4	14
LN(avg. CMD degree)	28	2.096	0.262	1.498	2.639
Jaccard index	28	0.844	0.168	0.333	1
Minimum cut	28	7.714	2.242	4	13
Minimum cut, normalized	28	0.863	0.136	0.444	1

^a^ This indicator includes individuals who were at least one year of age and who have not been away from the village for more than six months preceding the household survey. The number of people per household ranged from 1–15. The average number of people per household from those randomly sampled for the study is shown.

^b^ The estimate of the number of people per village is shown. The average household size for each village was multiplied by the total number of homes in each village. Amongst surveyed households, 9.64% (108/1120) of household heads owned more than one home, though it was not specified whether additional homes were within the same village of interest.

**Table 3:** Similarity of community medicine distributors.

Variables	Obs.	Proportion	Frequency
Direct tie	28	0.179	5
Selection similarity	28	0.536	15
Similarity in years as CMD	28	0.429	12
Age similarity	28	0.500	14
Gender similarity	28	0.429	12
Education similarity	28	0.464	13
Majority tribe similarity	28	0.464	13
Majority religion similarity	28	0.571	16
Occupation similarity	28	0.536	15
Formal status similarity	28	0.750	21
Similarity in use of protected drinking water sources	28	0.750	21
Similarity of private home latrine ownership	28	0.821	23
Home quality score similarity	28	0.750	21
Similarity in years settled in village	28	0.357	10

**Fig 1:**
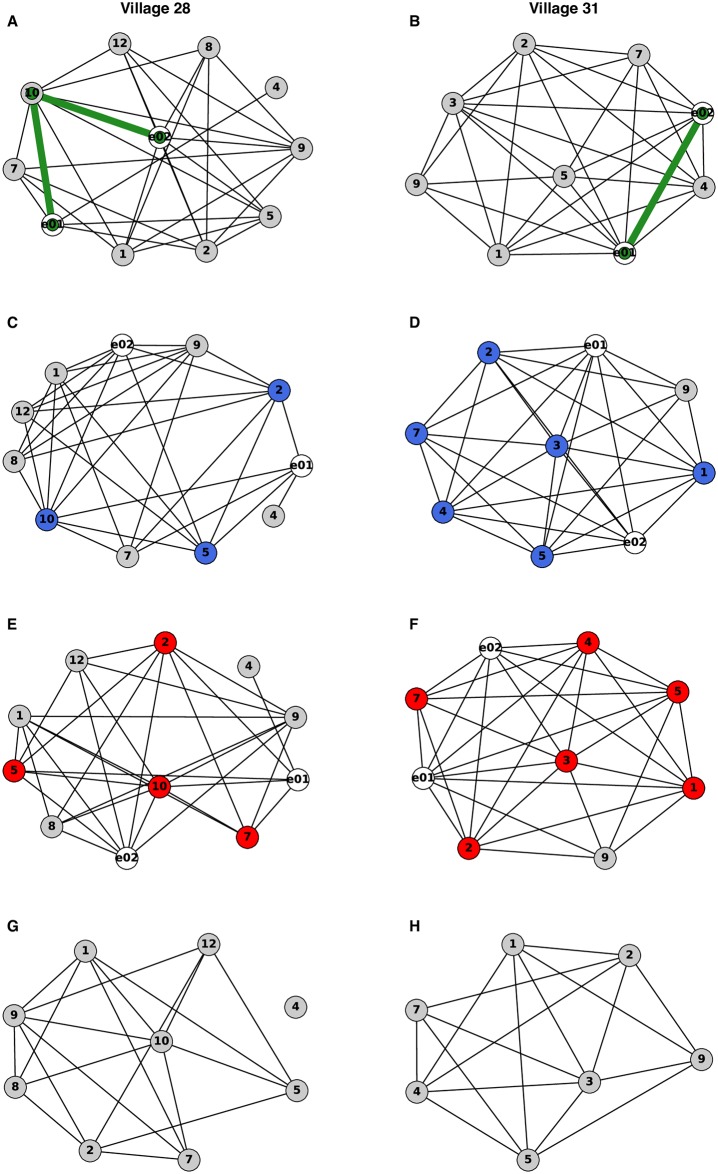
Network similarity. Real networks from two of the 28 study villages (IDs 28 & 31) are provided to illustrate the network similarity measurements. For each network, grey nodes represent the friends of CMDs; numbers correspond to the anonymous identifier of a friend within a particular village. White nodes and labels ‘e01’ and ‘e02’ are indicative of CMDs. The ties represent close friendships; all ties between CMDs and their friends as well as ties amongst friends of CMDs are shown. **A-B.** Green ties represent the shortest path between the two CMDs. In Village 31, there was a direct tie between CMDs, i.e. CMDs were close friends. In Village 28, only two ties separated CMDs, i.e. CMDs shared a common friend. For all study villages, CMDs were no more than two ties apart. For a village where CMDs were separated by two ties, there may be multiple shortest paths; only one shortest path is shown for illustration. **C-D.** Blue nodes represent the common friends between two CMDs within a village. **E-F.** Red nodes represent the minimum number of friends (nodes) that would need to be removed from the network to disconnect the two CMDs. The minimum node cut does not necessarily correspond to the number of common friends (e.g. village 28, panel C). **G-H.** These graphs show the friendships between friends of CMDs, after having removed both CMDs from the networks. Village 28 had a density of 0.528 and village 31 had a density of 0.810. There was only one village (ID 28) where the removal of CMDs resulted in a friend of the CMD (ID 4) becoming isolated from the CMDs’ network. Village 28 had the second lowest density amongst all the study village networks; the lowest being trivially different from the density of village 28 (0.526, village ID 29).

Network similarity between CMDs is illustrated in Fig 1 and summarized in Table 3. On average, each CMD had 8.43 friends. CMDs were close friends in only 5 of 28 villages (17.86%). Yet, CMDs did not belong to distinct friendship groups. Amongst the total number of friends named by both CMDs, an average of 84.40% of friends were shared between the two CMDs. Moreover, in every village, each CMD was no further than two steps apart, i.e. each CMD had at least one common friend. Over an average of seven friends had to be removed from the network to completely disconnect CMDs. The cohesiveness of CMDs was maintained through indirect ties because the friends of CMDs also were well connected. When both CMDs were removed from the network, density remained high amongst the friends. Here density is defined as the proportion of ties that exist amongst the maximum possible number of ties. The density amongst friends of CMDs was on average 0.784 (std. dev. 0.135, range 0.526–1). There was only one village (ID 28) where the removal of CMDs resulted in one friend becoming isolated from the network.

### Predictors of treatment & the division of labour

**Fig 2:**
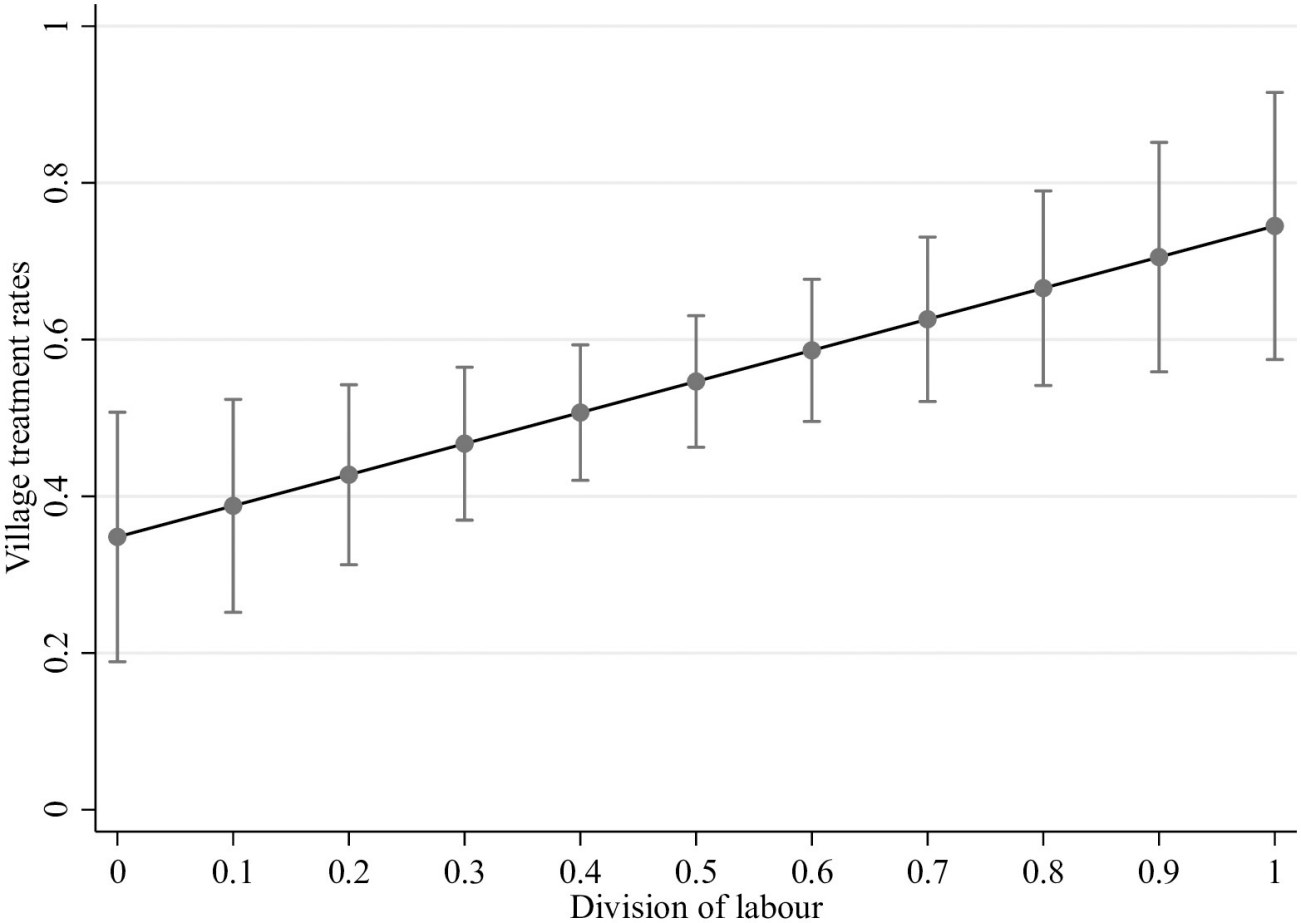
Predicted treatment rates by the division of labour. Both variables are measured at the individual level, i.e. for the percentage of eligible individuals offered at least one drug of praziquantel, albendazole, or ivermectin.

Table 4 presents the determinants of the percentage of individuals treated at the village level. Neither CMD similarity nor the sizes of the friendship networks and villages were associated with village treatment rates. Only one variable—the division of labour—was selected through LOOCV LASSO as a potential predictor of village treatment rates. The division of labour was positively correlated (p-value = 0.008) with village treatment rates. For the percentage of eligible individuals treated at the village level, there was a remarkable absolute difference of 39.69% between the treatment rates of CMDs with a perfect division of labour compared to CMDs with a perfectly unequal division of labour. The predicted village treatment rates against the range of values for the division of labour, i.e. the marginal effects of increasing equity in the division of labour, are shown in Fig 2. When the percentage of households treated was examined, the results for the division of labour were upheld despite LOOCV LASSO selecting two predictors in addition to the division of labour (Table 5). The discrepancies amongst village treatment rates at the household level for CMDs with and without equal divisions of labour were as large as 50.51% (p-value = 0.006). The additional predictors of the percentage of households treated included the similarity in the number of years spent as CMD and a village-level variable of the total homes. The total number of homes in the village was negatively related to village treatment rates at the household level (p-value = 0.001), although this effect was modest. A 10% increase in the total number of homes in a village was estimated to decrease the percentage of households treated by only 1.86%.

**Table 4:** Predictors of the percentage of eligible individuals treated.

Variable	Coef.	Robust SE	p-value	95% Confidence interval	
Division of labour	0.397	0.138	0.008	0.113	0.681
Constant	0.348	0.077	<0.001	0.189	0.507

Obs. 28

R^2^ = 0.176

*F-stat*. 8.25, p-value = 0.008

Root mean squared error (RMSE) from model LOOCV = 0.215

Variables selected through LOOCV LASSO.

Mean squared error (MSE) of LOOCV LASSO = 0.060

**Table 5:** Predictors of the percentage of households treated.

Variable	Coef.	Robust SE	p-value	95% Confidence interval	
Division of labour	0.505	0.167	0.006	0.160	0.850
Similarity in years as CMD	0.069	0.090	0.449	-0.117	0.256
LN(total homes)	-0.195	0.050	0.001	-0.298	-0.092
Constant	1.418	0.285	<0.001	0.830	2.005

Obs. 28

R^2^ = 0.393

*F-stat*. 13.18, p-value = <0.001

Root mean squared error (RMSE) from model LOOCV = 0.191

Variables selected through LOOCV LASSO.

Mean squared error (MSE) of LOOCV LASSO = 0.044

Tables 6 and 7 present the predictors of the division of labour between CMDs. For both individual and household level outcomes, the only predictor selected by LOOCV LASSO was the friendship between CMDs. A direct tie between CMDs was predicted to substantially increase the division of labour by 0.444 and 0.393, respectively, at the individual or household level when compared to CMDs without a direct tie. Hence, workload equity between CMDs was estimated to increase by just under twofold. Notably, the friendship between CMDs only predicted the division of labour and was not associated with village-level treatment rates (Tables 4 and 5, and *ρ* = 0.144, p-value = 0.464 at the individual level; and *ρ* = 0.098, p-value = 0.619 at the household level). There were no missed effects of other characteristics of CMD similarity influencing the division of labour due to indirectly affecting the presence of a direct tie (Table 8).

**Table 6:** Predictors of the division of labour (individual-level outcomes).

Variable	Coef.	Robust SE	p-value	95% Confidence interval	
Direct tie: friendship tie existed between CMDs	0.444	0.115	0.001	0.208	0.681
Constant	0.247	0.045	<0.001	0.155	0.340

Obs. 28

R^2^ = 0.393

*F-stat*. 14.91, p-value = 0.001

Root mean squared error (RMSE) from model LOOCV = 0.191

Variables selected through LOOCV LASSO.

Mean squared error (MSE) of LOOCV LASSO = 0.053

**Table 7:** Predictors of the division of labour (household-level outcomes).

Variable	Coef.	Robust SE	p-value	95% Confidence interval	
Direct tie: friendship tie existed between CMDs	0.393	0.126	0.004	0.134	0.652
Constant	0.257	0.050	<0.001	0.153	0.360

Obs. 28

R^2^ = 0.290

*F-stat*. 9.71, p-value = 0.004

Root mean squared error (RMSE) from model LOOCV = 0.213

Variables selected through LOOCV LASSO.

Mean squared error (MSE) of LOOCV LASSO = 0.063

**Table 8:** Spearman correlations with a direct tie between CMDs.

Variable	Rho	p-value
Jaccard index	0.088	0.656
Minimum cut, normalized	0.059	0.767
Selection similarity	-0.127	0.520
Similarity in years as CMD	-0.027	0.892
Age similarity	-0.093	0.637
Gender similarity	0.162	0.412
Education similarity	0.127	0.520
Majority tribe similarity	0.127	0.520
Majority religion similarity	0.027	0.892
Occupation similarity	-0.127	0.520
Formal status similarity	-0.162	0.412
Similarity in use of protected drinking water sources	0.054	0.786
Similarity of private home latrine ownership	0.217	0.267
Home quality score similarity	-0.162	0.412
Similarity in years settled in village	0.042	0.833
Total homes in village	0.162	0.411
Est. no. people per village	0.202	0.303

Obs. 28

## Discussion

In the context of rapidly expanding community-based MDA and WHO disease-specific goals of elimination [39–41], there is an urgent need to increase the effectiveness of CMDs. In accord with previous work [4], here we showed that treatment rates varied widely across villages in rural Uganda. The percentage of eligible individuals treated varied from 2.79–89.74% across 28 villages and worsened from an average of 59.05% per village in 2013 [4] to 47.77% in 2016 (our study). To gain a better understanding of how CMDs work and cooperate, we examined the influences of CMD similarity on the division of labour and village treatment rates.

The division of labour was highly unequal between CMDs, with one CMD treating on average only one third as many eligible individuals as the other CMD within the same village. The equality in the division of labour was positively associated with overall village treatment rates. The estimated difference between a perfectly equal (a 50–50 split of individuals treated) and unequal (one CMD treating no one) division of labour was remarkable. An equal division of labour was associated with the treatment of an additional 39.69% more of the eligible population or 50.51% more households approached when compared to an unequal division of labour. Considering that WHO-recommended treatment rates for effective morbidity control are 65% for lymphatic filariasis and 75% for schistosomiasis or STHs, our results suggest that treatment targets are not achievable without an equitable distribution of labour between CMDs. These findings also highlight that a discussion of CMD capacity constraints [14] is unfounded within villages where one CMD treats few or no people. It is unknown whether adding more volunteer CMDs would facilitate MDA or simply contribute to an even more unequal division of labour and idle labour. Efforts to reduce CMD attrition rates [42] may be counterproductive if the result is that poorly performing CMDs are retained [21]. National MDA programmes should focus on the quality rather than the numbers of CMDs. Characteristics that may be used to improve the selection of CMDs can be gleaned from hardworking CMDs—defined here as CMDs who treated many individuals. In our study area, hardworking CMDs have been shown to be individuals who engage in good preventative health behaviours, belong to high-risk groups for endemic NTDs (e.g. fishermen for schistosomiasis), are male, and have supportive friendship networks [4, 21]. Ultimately, the selection/replacement criteria for CMDs should align with factors that are of interest to NTD-endemic communities.

The only determinant of the division of labour was a direct tie, i.e. close friendship between CMDs. Friendship was neither indicative of contact between CMDs nor of who knew whom. CMDs belonged to villages that were small with respect to population size and geographical spread. The variation in village size (total homes and total population) was uncorrelated to the presence of a direct tie. This result might suggest that village size also was unrelated to the frequency of contact between CMDs, assuming that the presence of a direct tie was partially determined by the frequency of in-person contact. CMDs knew each other well; they were both selected by individuals within the same village, trained together annually for MDA, and shared many common friends. Hence, CMDs had similar social networks. Yet, few villages (5/28) had CMDs who themselves were friends. Therefore, improving the division of labour is not as trivial as introducing two CMDs. Communication between CMDs was essential to improving the equity in the distribution of work related to MDA [28]. Here we only examined the ties between CMDs within the same village. Future research is needed to understand how CMDs are connected across villages. National MDA programmes do not hold regular meetings to bring together CMDs apart from the annual training. Encouraging CMDs both within and perhaps across villages to meet more frequently—maybe monthly—to compare and submit data, collect additional MDA supplies (registers, medicines, etc.), and simply to socialize may lead to new friendship connections that could facilitate communication between CMDs.

CMD friendship was only associated with village treatment rates indirectly, i.e. through the division of labour. This finding accords with previous research that tracked MDA in our study area [4] and found that a friendship tie between CMDs was not directly correlated with village treatment rates. Surprisingly, similar CMDs were not more likely to be friends than CMDs with different attributes. Conventional wisdom on social networks [23] suggests that direct ties exist between individuals in part due to homophily, which is the tendency of individuals to connect with others most like themselves. However, here we showed that shared socioeconomic characteristics did not predict the presence of a direct tie between CMDs. The presence of a direct tie is one indicator of CMDs belonging to the same cluster within the broader village social network. Stifling of an intervention hypothetically could occur by trapping its spread (information or uptake) within a confined set of closely-knit, clustered individuals [43]. There was no support that the reach of MDA was stifled when two CMDs were within the same network cluster. In contrast, we found that implementing MDA with CMDs in the same network cluster indirectly was positively associated with village treatment rates by improving the division of labour. It remains an open question as to whether negative ties (active dislike) exist between CMDs who are not close friends. The positive effect of direct ties on the division of labour suggests that CMDs are complements rather than substitutes with respect to their labour input. Complementary inputs result in additive or multiplicative effects on cooperation and equality between CMDs whereas substitutes might suggest a crowding out effect of one CMD working harder thereby causing the other CMD to work less. We cannot rule out that CMDs planned their division of labour, perhaps trading off efforts where one CMD agrees to take on the majority of responsibility for MDA this year whilst the other CMD resumes duties in the following year. If CMDs negotiated the division of labour then we would expect a direct tie, which represents a channel of communication between CMDs, to be negatively related to village treatment rates. Yet, no such association was observed. There remains the possibility of a motivational imbalance between CMDs [19] that is unrelated to CMD similarity. Regardless of the reason for a highly unequal division of labour, this inequality undermined community-based MDA and was correlated with low village treatment rates.

Our definition of the division of labour captured a number of features relevant to our study area and similar contexts. There were two CMDs. MDA was conducted in rural, small villages that did not have further geographical sub-units. There were no MDA programme stipulations for if/how labour between CMDs should be divided. Importantly, our measurement of the division of labour was outcome-based, i.e. dependent on the number of people to be treated. This definition is in light of WHO treatment targets and thus, from the perspective of the agency rather than from the view of the community. To investigate the division of labour in other MDA contexts, there is a need to develop a typology of the division of labour that includes a range of options for dividing the population as well as methods for evaluating labour/effort from MDA volunteers.

A provisional typology of the division of labour might include, as studied here, the number of people to be treated or instead could be focused on tasks, community geography, or social groups. CMDs performed MDA tasks of registering households, sensitizing individuals, and mobilizing the community whilst administering treatment instead of before treatment as instructed by the national programmes [4, 13, 20]. Thus, in our study area, there was no distinction between tasks and treatment. However, it remains an open question as to if creating a distinction between tasks and treatment, and dividing by task increases CMD productivity. Community participation is needed to understand the value of each MDA task and to identify tasks that are conducted by CMDs and the wider community but not recognized by national MDA programmes. The identified tasks might be enumerated in a form that is used to monitor CMDs by both national programmes and NTD-endemic communities. Beyond MDA, CMDs are members of the village health team, which is responsible for a wide range of primary health care tasks including bed net distribution, the management of childhood illnesses, and individual referrals to government health services [44]. There is a need to better understand the process in which CMDs divide responsibilities for MDA tasks versus other primary health care activities. In our study, we did not have information on whether CMDs who treated no one during MDA were actively engaged in other primary health care interventions.

Although village size did not affect the division of labour in our study area [4], spatial dimensions, population size, and local ecology will need to be considered in urban or peri-urban settings. A system could be devised using local knowledge of ecological or administrative divisions to assign the population to be treated. Local knowledge is critical, as—anecdotally in our study district—government records of villages did not accord with existing villages. This misalignment was not due to inaccuracies in reporting but rather the quickly change shape of local boundaries as determined by the communities themselves.

Potential social groups that could be used to divide labour include kinships, friendship groups, gender, occupations, burial societies, saving cooperatives, tribes, clans, or religious associations. In our study area, both kinships [16] and friendship networks [4, 13] have been shown to affect treatment rates, whereas gender has not been a barrier to who treats whom [21]. Women and men are just as likely to treat individuals from another gender when compared to how many people they treat of the same gender [21]. We found no support that similarities/differences in demographic or socioeconomic characteristics affected the division of labour or village treatment rates. This result was surprising in that social imbalances did not lead to a highly unequal division of labour, i.e. there was no evidence that high status CMDs were free riding off the efforts of lower status CMDs [13]. Diversity between CMDs did not translate into higher village treatment rates [16]. Villages with CMDs who represented more social groups, assuming CMD attributes were indicative of social group memberships, did not achieve higher village treatment rates than villages with CMDs from the same social groups. Although CMD diversity did not translate into higher village treatment rates, future work should examine whether CMD diversity affects the treatment rates of marginalized, underrepresented populations [20]. The usefulness of social divisions for the treatment of marginalized populations will depend foremost on the homogeneity of the communities to be treated and personal attributes of CMDs. Homogenous populations will not have natural social divisions. Additional research is needed to 1) develop indicators for assessing the variation of homogeneity between NTD-endemic communities, 2) measure if/how homogeneity impacts treatment rates, 3) develop methods to ‘match’ CMDs with individuals to be treated using a wide range of socioeconomic characteristics, and 4) analyze how aligning CMDs with similar social groups affects their treatment rates.

Developing a typology for dividing labour is only the first step in fully evaluating CMD equity. A limitation of our study is that we did not directly assess the effort expended by CMDs. For example, we require an understanding of how difficult it is to complete a particular task or traverse different terrains. The number of hours spent per task has been enumerated elsewhere from the perception of CMDs [14]. If time constraints were determined by occupation or familial demands (related to age and gender) then we would expect differences in CMD attribute similarity to capture differences in CMD time constraints. In that case, our results might suggest that differences in time constraints between CMDs did not explain the division of labour. Additional studies are needed to assess differences in time spent on MDA versus other primary health care tasks and the effect on the division of labour and MDA treatment rates. All CMDs received the same remuneration for attending MDA training and no other payments from MDA programmes. However, CMDs who engage in other primary health care tasks may be remunerated and these additional sources of income might affect CMD effort during MDA. To identify indicators in addition to time and remuneration for evaluating effort, community expectations for CMDs also should be examined.

Improving the division of labour between CMDs is a social problem. The Declaration of Alma-Ata in 1978 emphasized the need to account for the social determinants of treatment and disease [45]. Our study emphasizes the importance of the message of Alma-Ata for community-based MDA. Social relations between CMDs should be improved and, to do so, community involvement in MDA must be increased. The shift in terminology from ‘community-directed’ MDA, which originated in the 1970s, to the now widely used ‘community-based’ MDA suggests a reduction in the role of NTD-endemic communities [46]. Reducing the role of communities from actively designing/monitoring treatment programmes (community-directed) to nominating CMDs (community-based) risks turning communities into passive actors during MDA. Even a slight deviation from community-based MDA towards community-directed MDA, whereby CMDs involve their close friends in monitoring or disseminating information, has been associated with increased treatment rates [21]. To move from passive to active community involvement, there is a need to reimagine the concept of equity as posed in this study. Here we examined the equity between CMDs in light of their ability to meet programmatic goals (treatment targets). Equity instead could be explored by examining to what extent CMDs and communities, or communities and the national programme are equal partners in designing and running MDA. An ultimate goal for MDA might be the integration into local health systems to empower communities to manage their own health [46].

To improve the effectiveness of community-based MDA, national programmes should facilitate the equal division of labour between CMDs. The similarity of personal attributes of CMDs was unrelated to the best combination of CMDs with the most equal division of labour and, in turn, highest treatment rates. National MDA programmes may instead aim to foster friendships between CMDs or to encourage the selection of CMDs who already are close friends in order to promote open communication between CMDs. Alternatively, guidelines might be trialed where an equal division of labour is encouraged between CMDs; treatment rates may be recorded for each CMD and monitored to identify inactive CMDs. Future interventions also might seek to explore other avenues to increase community involvement in the design of MDA. A more equal division of labour between CMDs may assist NTD programmes with achieving treatment targets.

## Supporting information

S1 File Strobe checklist. (DOC)

S1 Data Study data. (XLS)
